# Nitrogen Acquisition Strategies Mediated by Insect Symbionts: A Review of Their Mechanisms, Methodologies, and Case Studies

**DOI:** 10.3390/insects13010084

**Published:** 2022-01-12

**Authors:** Xueming Ren, Ruxin Guo, Mazarin Akami, Changying Niu

**Affiliations:** 1 Hubei Key Laboratory of Insect Resource Application and Sustainable Pest Control, College of Plant Science & Technology, Huazhong Agricultural University, Wuhan 430070, China; ren_xueming@126.com (X.R.); gracie27@163.com (R.G.); makami1987@gmail.com (M.A.); 2 Department of Biochemistry, Faculty of Science, University of Douala, Douala 24415, Cameroon

**Keywords:** insect symbionts, biological nitrogen fixation, nitrogenous waste recycling, GS/GOGAT cycle, amino acid biosynthesis

## Abstract

**Simple Summary:**

Nitrogen acquisition strategies mediated by insect symbionts through biological nitrogen fixation (BNF) and nitrogenous waste recycling (NWR) were reviewed and compared in our paper, and a model for nitrogen provisioning in insects was then constructed. In our model, (1) insects acquired nitrogen nutrition from food stuffs directly, and the subprime channels (e.g., BNF or NWR) for nitrogen provisioning were accelerated when the available nitrogen in diets could not fully support the normal growth and development of insects; (2) the NWR strategy was more accessible to more insects due to its energy conservation and mild reaction conditions; (3) ammonia produced by different channels was used for essential nitrogenous metabolites synthesis via the glutamine synthetase and glutamate synthase pathways.

**Abstract:**

Nitrogen is usually a restrictive nutrient that affects the growth and development of insects, especially of those living in low nitrogen nutrient niches. In response to the low nitrogen stress, insects have gradually developed symbiont-based stress response strategies—biological nitrogen fixation and nitrogenous waste recycling—to optimize dietary nitrogen intake. Based on the above two patterns, atmospheric nitrogen or nitrogenous waste (e.g., uric acid, urea) is converted into ammonia, which in turn is incorporated into the organism via the glutamine synthetase and glutamate synthase pathways. This review summarized the reaction mechanisms, conventional research methods and the various applications of biological nitrogen fixation and nitrogenous waste recycling strategies. Further, we compared the bio-reaction characteristics and conditions of two strategies, then proposed a model for nitrogen provisioning based on different strategies.

## 1. Introduction

Nitrogen is an essential nutrient for insects, largely required for building cells, tissues, and life molecules [1]. Generally, insects acquire nitrogen from diet to maintain their internal nitrogen balance. However, for many insects living in the low nitrogen niches, especially herbivores, the low content of total available nitrogen and the unbalanced supply of essential amino acids (EAAs) in diets severely limit their growth and development [2,3]. Furthermore, large quantities of nitrogenous metabolites are excreted with feces throughout insects’ life history, which intensifies the low nitrogen stress [1,4]. Insects cannot compensate for extremely low dietary nitrogen by overeating ad infinitum, nor could they synthesize essential metabolites directly from inorganic nitrogen. As such, nutritional provisioning by symbionts seems to be an important way for insects to avoid nitrogen starvation [4].

Nitrogen acquisition strategies mediated by insect symbionts mainly include nitrogen enrichment, biological nitrogen fixation (BNF) and nitrogenous waste recycling (NWR) [5,6,7]. Among them, nitrogen enrichment is a relatively simple manner, in which symbionts act as food substrate and consumed by insects directly. For example, fungus gardens cultivated by *Odontotermes formosanus* provide termite workers 93.0% of α-amino acids and fatty acids. Notably, the fungal nodules, which are spherules growing on fungus gardens, exclusively produce tryptophan for termites [8]. BNF and NWR, functioned in insect–symbiont complexes, are more efficient and prevalent in nature and play crucial roles in nitrogen assimilation. Here, we systematically summarized the reaction mechanisms, conventional research methods and application cases of these two strategies in insects, aiming to better understand the highly efficient nitrogen economy existing in insect–symbiont complexes.

## 2. Reaction Mechanisms of Nitrogen Acquisition Strategies Mediated by Symbionts

### 2.1. Biological Nitrogen Fixation

**Figure 1:**
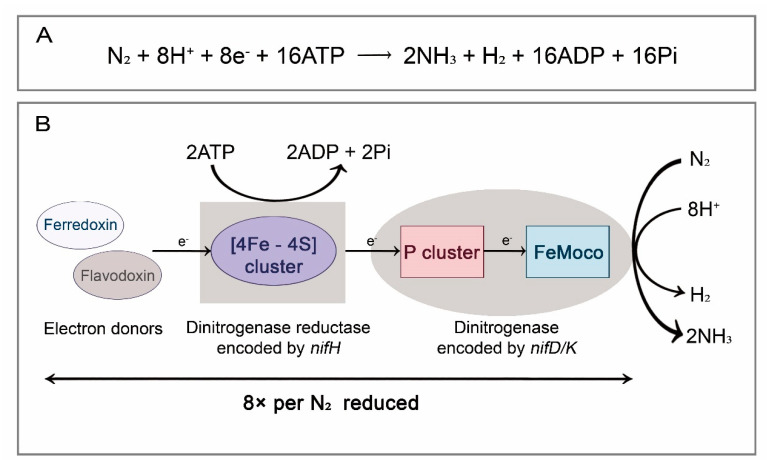
BNF reaction and its molecular mechanism. (**A**) Chemical reaction formula for BNF; (**B**) schematic diagram of dinitrogen reduction. Electrons are transferred from ferredoxin/flavodoxin via dinitrogenase reductase to dinitrogenase. At least 16 molecules of MgATP are consumed for each reduction of a dinitrogen.

Prokaryotes contribute to approximately 176 million tons of nitrogen fixation per year in terrestrial and marine ecosystems, accounting for 67.7% of the total global nitrogen fixation, while in arthropod intestines, organic nitrogen fixed by diazotrophic bacteria is about 10–40 kg/ha/year [9]. The classical BNF reaction is catalyzed by nitrogenase complex (Mo/Fe nitrogenase), which consists of two main functional subunits: dinitrogenase reductase (γ_2_ homodimeric azoferredoxin, encoded by *nifH* gene) and dinitrogenase (α_2_β_2_ heterotetrameric molybdoferredoxin, encoded by *nifD* and *nifK* genes). Neither dinitrogenase reductase nor dinitrogenase shows nitrogenase activity separately. The reduction of atmospheric nitrogen to ammonia is catalyzed only if they form a complex with each other. In this reaction, the [4Fe-4S] cluster on dinitrogenase reductase accepts electrons provided by electron donors (such as ferredoxin and flavodoxin) and transfers electrons to the metal atom center (P clusters) on dinitrogenase. The P cluster (an [8Fe–7S] cluster) is an electron transport intermediate, and is responsible for accepting and storing electrons from the [4Fe-4S] cluster and transferring them to the FeMo cofactor (FeMoco), where dinitrogen receives electrons, thereby producing ammonia and releasing H_2_ (Figure 1) [10,11,12].

In addition to Mo/Fe nitrogenase mentioned above, two other genetically distinct nitrogenase metalloenzyme systems, but clearly homologous with Mo/Fe nitrogenase, are known in the BNF process: Fe/V nitrogenase and Fe/Fe nitrogenase. Fe/V nitrogenase is encoded by Vnf gene cluster (*vnfD*, *vnfG*, *vnfH*, *vnfK*), whose metal center is composed by iron and vanadium; Fe/Fe nitrogenase is encoded by Anf gene cluster (*anfG*), whose metal center is only composed by iron [11,13]. The nitrogen fixation efficiency of Fe/Mo nitrogenase is about 1.5 times than that of Fe/V nitrogenase at 30 °C, and the efficiency of Fe/Fe nitrogenase is the lowest [14]. To date, all reports about BNF strategies in insects rely on the Fe/Mo nitrogenase metalloenzyme systems. Fe/V nitrogenase has only been reported in *Azotobacter vinelandii* and *A. chroococcum*, while Fe/Fe nitrogenase has only been demonstrated in *A. vinelandii* and *Rhodobacter capsulatus* [12].

### 2.2. Nitrogenous Waste Recycling

Traditional NWR strategy refers to the utilization of animal nitrogenous metabolites by symbionts to synthesize EAAs, which are translocated back to the animal host [15]. For insects, the type and relative content of nitrogenous compounds are closely related to their living environments and diets, and vary greatly among different insect species. Generally, nitrogenous wastes of terrestrial insects are dominated by uric acid, which is of great significance for maintaining moisture in their tissues [16,17]. However, water retention is not very important for aquatic insects, so ammonia/urea is excreted mixed with feces to reduce the toxicity of ammonia to cells. Interestingly, some insects, such as *Sialis* and *Dytiscus*, whose larval stage live in water but adults live in land, gradually change the main component of their excrement from ammonia to uric acid as the larvae grow into adults. In addition, there are other types of nitrogenous metabolites excreted by a few insects, including allantoin, allantoic acid, as well as protein, xanthine, hypoxanthine, teropterine, creatinine, etc. [18,19].

**Figure 2:**
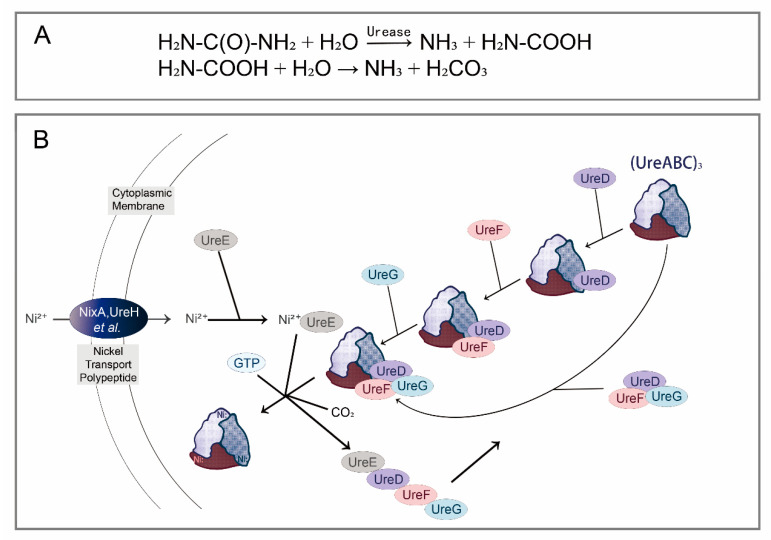
Urea hydrolysis reaction and molecular mechanism diagram of urease activation. (**A**) Chemical reaction formula for urea hydrolysis; (**B**) urease activation model in vivo. The structural proteins encoded by *ureA/B/C* constitute an inactive apoprotein, denoted as (ureABC)_3_, whose activation in vivo requires the participation of Ni^2+^, CO_2_, GTP, and numerous urease accessory gene products. *ureD*, *ureF*, and *ureG* sequentially combine with (ureABC)_3_ to form a ureABC–ureDFG complex. Alternatively, *ureD*, *ureF*, and *ureG* first form an ureDFG heterotrimer, and then combine with (ureABC)_3_. After that, the active sites on the ureABC–ureDFG complex can bind to Ni^2+^ delivered by *ureE* accessory proteins. Carbon dioxide is used to form the carboxy-lysine metal ligands; GTP hydrolysis (occurring in *ureG*) powers the assembly of the metallocenters, drives the activation of urease, and releases all accessory proteins (involved in the next urease activation process, subsequently). This reaction finally forms three catalytic sites on the urease, each containing two Ni^2+^.

The bio-reaction pathways of different nitrogenous wastes reabsorption are the same (KEGG: M00546). In this process, urea, as a downstream metabolite, could be hydrolyzed by urease, producing carbon dioxide and ammonia (reabsorbed by insect–symbiont complexes eventually) [20]. Urease, encoded by urease gene cluster (*ure*), is a nickel-containing oligomerase, mainly including structural proteins (encoded by *ureA*, *ureB*, and *ureC*) and auxiliary proteins (encoded by *ureD*/*ureH*, *ureE*, *ureF*, *ureG*, and *ureI*) [21,22]. Structural genes *ureA*, *ureB*, and *ureC* encode γ, β and α subunit of urease, respectively, forming a complex with inactive apourease; and nickel ions are then transported to inactive apourease driven by auxiliary proteins to make it functional (Figure 2). In addition to structural and auxiliary genes, some regulatory genes are also reported in urease gene clusters; for example, *ureR* gene in *Proteus mirabilis*, *Providencia stuartii*, *Escherichia coli*, and *Salmonella* encodes a positive regulator similar to cytarabine (AraC), which promotes the expression of urease when urea is present. The activity of urease in symbionts is not only regulated by regulatory genes, but regulated by environmental factors, such as hydrolysis product concentrations (i.e., NH_3_ and nitrogen levels), nutritional status, pH value, and urea concentrations [21,23].

Adding a moderate amount of urea to diets for substituting expensive animal or vegetable-derived protein sources has been widely applied in ruminants breeding for many years [24,25]. In ruminants, 40 to 80% of endogenously produced urea-N is returned to the gastrointestinal tract, and this part of urea is an important source of nitrogen for microbial protein synthesis [24]. However, excessive dietary urea or high urease activity leads to the accumulation of ammonia in ruminants, causing gastrointestinal lesions and poor nutrient absorption [26]. Hence, reasonable adjustment of the dietary urea content or urease activity in the rumen of ruminants is the key to improve the efficiency of urea utilization and reduce the risk of ammonia poisoning in animals [21,27]. Some preliminary attempts have been made to use urea or ammonia in insect rearing. For instance, urea in low nitrogen diets could be absorbed by *Bactrocera oleae*, which significantly elevate the fecundity of females [28]; organic waste to biomass conversion ratio can be significantly improved with the addition of *Rhizopus oligosporus* and ammonia when using *Hermetia illucens* for treatment of wastes [29].

Ammonia generated via BNF and NWR is firstly incorporated into glutamine and glutamate under the catalysis of glutamine synthetase (GS) and glutamate synthase (GOGAT) enzyme complex (i.e., GS/GOGAT cycle). Glutamate is then involved in nitrogen metabolism, and synthesized EAAs (valine, leucine, isoleucine, histidine, methionine, threonine, lysine, phenylalanine, tryptophan, and arginine) [20,30].

## 3. Conventional Methods Related to BNF and NWR in Insects

### 3.1. Target Species Isolation and Functional Genes Identification

Enrichment and purification of target strains using selective culture medium is still a conventional technique at the present stage. For example, Dobereiner nitrogen-free medium is usually used in the nitrogen-fixing bacteria isolation [31,32]. However, a few bacteria which have a low nitrogen requirement can also grow and proliferate well on nitrogen-free medium, so functional genes analysis is necessary for the identification of nitrogen-fixing bacteria. The *nifH* gene is usually used for the analysis of phylogenetic diversity and classification of diazotrophic bacteria in samples due to its wide distribution and high conservation [33,34]. In addition to the encoding genes of nitrogenase (i.e., *nifH*, *nifD* and *nifK*), three accessory genes *nifE/N/B*, involved in FeMoco synthesis and nitrogenase maturation, are also present in most diazotrophs [35]. Hence, a new criterion proposed in a study for computational prediction of nitrogen fixation is to use the presence of the *nifH/D/K* and *nifE/N/B* genes [34,35,36].

Culture medium, in which nitrogenous metabolite as the main nitrogen source, is usually used in screening target bacteria mediating NWR strategy. For example, Christensen agar base (20 g of urea/L, 1 g of Peptone/L) is specifically used for urease-positive bacteria isolation [37]. Similar to the *nifH* gene in diazotrophic bacteria, the *ureC* gene encoding the main functional subunit of urease possesses multiple highly conserved regions [21,24] and is widely present in ureolytic bacteria (*Helicobacter Pylori* contains only *ureA* and *ureB*) [38,39]. Therefore, *ureC* gene is often designed as a primer pair for ureolytic bacteria identification in different environments.

### 3.2. Enzyme Activity Determination

#### 3.2.1. Determination of Nitrogenase Activity

Acetylene reduction assay (ARA) has been commonly used in the laboratory and in the field for the last 50 years for determining the activity of nitrogenase [36]. Nitrogenase catalyzes the reduction of acetylene to ethylene under favorable conditions, then ethylene can be detected by gas chromatography. The amount of nitrogen fixed by diazotrophs is calculated according to the molar ratio of ethylene: nitrogen = 4:1 or 3:1 [40,41]. The protein produced by BNF was predicted based on the fact that the nitrogen content in protein accounted for about 16%. According to this algorithm, the nitrogen equivalents of 6 µg of protein were fixed per fly per day in *Ceratitis capitata* [42]; about 0.25 µg of nitrogen were fixed per larva per day in *Dorcus rectus* [43].

#### 3.2.2. Determination of Urease Activity

Urease, a strict intracellular enzyme, converts urea to ammonia, which leads to an increase in the pH of the culture broth [37]. Qualitative urease tests were performed by adding an appropriate acid–base indicator (such as phenol red) into the broth and then observing the change in color within two hours; and quantitative urease assays were performed by determining the rate of ammonia produced from urea hydrolysis [44,45]. The results of quantitative urease assays are commonly expressed as micromoles of ammonia produced per minute per milligram of urease protein. In addition, enzyme-linked immunosorbent assay (ELISA) is also suitable for quantitative urease assays [46]. In this way, the absorbance of final product is proportional to the concentration of urease protein at a particular wavelength, so the concentration of urease in samples could be calculated based on the standard curves of urease concentration-absorbance.

### 3.3. Isotopic Tracer Technique

The presence of functional strains and genes is the prerequisite for insects to initiate BNF or NWR strategy, but it does not necessarily indicate that insect–symbiont complexes can successfully produce ammonia in vivo, because these enzymatic processes are highly regulated by many factors [5,10]. Further, even though ammonia can be produced by nitrogenase or urease in vivo, it does not promise to be assimilated and utilized by insects. To compensate for the shortcomings of the above methods, the isotope tracer technique has been exploited for studying the nitrogen cycle processes. Usually, samples are placed in an airtight container, in which the N^15^-labeled atmospheric nitrogen is injected moderately. After that, using isotope mass spectrometer to detect the content of N^14^ and N^15^ in samples within a certain time and calculate the values of δ^15^N, which reflects the efficiency of nitrogen fixation [47]. Similarly, N^15^-labeled nitrogenous waste compounds, such as uric acid [48] and urea [30,49], are usually added to the diets when evaluating the efficiency of NWR, the values of δ^15^N in samples are calculated after feeding a period of time.

Calculation formula for δ^15^N [30,50]:

δ = 1000 [{(^15^N_sample_/^14^N_sample_)/(^15^N_standard_/^14^N_standard_)} − 1] (1)

### 3.4. High-Throughput Sequencing

The technology of high-throughput sequencing greatly facilitates the analyses of strains composition and diversity, and provides technical support for predicting the functions of uncultured strains [6,51]. Phylogenetic and functional gene amplicons is the gold standard for analyzing the diversity and composition of microbial communities in samples [52]; metagenomics and metatranscriptomics sequencing can not only reveal the composition profile and expression level of all microorganisms deeply, but also help us with metabolic pathways construction and gene expression analysis [53]. The complete genome and transcriptome sequencing of a single strain are helpful for the study of functional genes diversity and the prediction of new functions in this strain. Currently, high-throughput sequencing has been widely used in studying the nitrogen cycle in the environment and has presented many novel interactive models between insects and symbionts [20,54,55].

## 4. The Various Applications of BNF and NWR Strategies in Insects

### 4.1. Application Cases of BNF Strategy in Insects

A recent review paper showed that potential diazotrophs widely exist in Coleoptera, Diptera, Hemiptera, Blattaria, Hymenoptera, Lepidoptera, and Thysanoptera [5]. Nitrogen fixation has been extensively studied and convincingly demonstrated in cockroaches and termites. Besides this, BNF strategy can also be exploited by other insects for nitrogen provisioning, mainly including the longhorned beetle (e.g., *Anoplophora glabripennis*, *Prionoplus reticularis*), the weevil (e.g., *Conorhynchus palumbus*), the bark beetle (e.g., *Dendroctonus rhizophagus*, *D. valens*), the click beetle (e.g., *Agriotes obscurus*, *Selatosomus aeneus*), the stag beetle (e.g., *D. rectus*), and the bess beetle (e.g., *Odontotaenius disjunctus*) in Coleoptera; tephritid fruit flies (e.g., *Ceratitis capitata*, *Bactrocera tryoni*) in Diptera; the carmine cochineal (e.g., *Dactylopius coccus*, *D. opuntiae*) in Hemiptera; and leaf-cutter ants (e.g., *Acromyrmex echinatior*, *A. volcanus*, *A. octospinosus*) and sirex wood wasp (e.g., *Sirex noctilio*) in Hymenoptera.

BNF-related symbionts are not limited to the intestinal tract of insects. For instance, the carmine cochineal endosymbiont *Dactylopiibacterium* attaches to the surface of host ovary for vertical transmission, and expresses nitrogenase activity in host hemolymph and ovary [56]. Furthermore, BNF reaction occurred in vitro, such as in the immediate living environment of insects or insect-associated organisms, can also benefit to insect nitrogen budget. For example, the tunnels of some xylophagous insects, the nests of termite, or the fungus gardens cultivated by leaf-cutter ants fill with large quantities of frass and food residue, which act as preferred substrate for diazotrophs. Nitrogen nutrition in frass and food residue fixed by diazotrophs can be consumed by insects directly [50,57,58].

### 4.2. Application Cases of NWR Strategy in Insects

Three alternative scenarios of symbiotic NWR, different in their patterns of amino acid synthesis using waste ammonia, may be functional in the symbiosis between insects and symbionts [15]: (A) Symbiont-mediated: Symbionts convert nitrogenous waste compounds of insects into EAAs for host assimilation and absorption; (B) Mediated by host cell–symbiont complex: Ammonia is firstly assimilated into non-essential amino acids (nEAAs, such as glutamine and glutamate) by GS/GOGAT in insect cells. nEAAs are used as ammonia donors to synthesize EAAs in symbionts; (C) Host cell-mediated: Ammonia is assimilated into glutamate by GS/GOGAT in insect cells. At the same time, intracellular symbionts synthesize carbon skeleton of EAAs and secrete them into insect cells. EAAs are assembled in insect cells catalyzed by transaminases. These NWR patterns could be employed by insects in Blattaria, Coleoptera, Diptera, Hemiptera, and Hymenoptera for nitrogen provisioning (Table S1).

#### 4.2.1. Blattaria

Nitrogenous wastes in cockroaches (e.g., *Periplaneta* *americana*, *Blattella germanica*) are stored in their fat bodies in the form of uric acid. When dietary nitrogen is limited, endosymbiont *Blattabacterium cuenoti* inhabited in the fat bodies of cockroaches synergizes with the host to degrade uric acid and assimilate the degradation products into EAAs. In this process, cockroaches synthesize urate oxidase, allantoinase, and allantoicase, which are necessary for uric acid degradation, and supply nEAAs to *B. cuenoti*. *B. cuenoti* recycles nitrogen from urea and ammonia into glutamate under the catalysis of urease and glutamate dehydrogenase, and then synthesizes all the EAAs, various vitamins, and other required compounds for the host insect [54,59,60]. During the evolution of cockroach to termite, *B. cuenoti* was gradually lost from the fat body, and its metabolic functions were replaced by the symbiotic flora in termite hindgut. Therefore, uric acid in termites (e.g., *Reticulitermes flavipes*) needs to be transferred to hindgut through malpighian tubules before it can be degraded by gut symbionts [61,62]. *Mastotermes darwiniensis* is the only lower termite that retains the endosymbiont *B. cuenoti*. However, compared with *B. cuenoti* in cockroaches, the genome of *B. cuenoti* in *M. darwiniensis* is sharp reduced, so it is speculated that uric acid degradation in *M. darwiniensis* should be co-mediated by *B. cuenoti* and hindgut symbionts [63,64]. Uricolytic strains, such as *Clostridia*, Enterobacteriaceae, also widely exist in the guts of *Reticulitermes speratus*, *Coptotermes formosanus*, *Neotermes koshunensis*, *Glyptotermes fuscus*, *Cryptotermes domesticus*, *Hodotermopsis sjoestedti*, *O. formosanus*, and *Nasutitermes takasagoensis*. It is estimated that an amount of uric acid nitrogen equivalent to 30% of the total nitrogen in an average termite colony may be recycled or redistributed annually through the action of gut uricolytic bacteria [16].

#### 4.2.2. Coleoptera

The levels of uric acid in bark beetles (*D. rhizophagus*, *D. valens*) are significant different in whole eggs, larvae, and adults (male and female). Among them, the highest uric acid content is detected in female adults, whose guts contain various uricolytic bacteria, such as *Pseudomonas fluorescens*, *Serratia proteamaculans,* and *Rahnella aquatilis* [65]. Urea-hydrolyzing bacteria present in the egg surface and larval guts of *Anoplophora glabripennis* or the larval and adult guts of *Melolontha hippocastani* can incorporate nitrogen from ingested urea back into the insect tissues [30,49].

#### 4.2.3. Diptera

As mentioned above, adding urea or ammonia to artificial diets can significantly improve the female fecundity of olive fruit fly and the larval biomass of black soldier fly. In addition, the bacterium *Enterobacter agglomerans* isolated from the alimentary tract of the apple maggot fly, *Rhagoletis pomonella*, mediates purine (such as uric acid) degradation, and the degradation products were significantly attractive to *Anastrepha ludens* and *R. pomonella* [66,67].

#### 4.2.4. Hemiptera

Most plant sap-feeding hemipteran insects feed on diets with an extremely low or unbalanced nutrient content. Therefore, many plant sap-sucking insects (such as psyllids, whiteflies, mealybugs, aphids, cicadas, spittlebugs, and sharpshooters) rely on obligatory endosymbionts with much-reduced genome size to synthesize B vitamins, steroids, EAAs, and other nutrients [51,68,69,70]. The application of NWR strategy in hemipteran insects has been verified in shield bugs, brown planthoppers, cochineal insects, aphids, and others. *Erwinia*-like bacteria, vertically transmitted through eggs, are widely present in the midgut of stink bugs and they synthesize uricase, allantoinase, and allantoicase in the cecum of *Parastrachia japonensis* to catalyze the degradation of uric acid. Uric acid is recycled for EAAs syntheses with the aid of *Erwinia*-like bacteria, thereby leading to significant improvement of the survival rate of adults and nymphs [18]. Different from *P. japonensis*, *Nilaparvata lugens* on its own is capable of encoding partial uricolytic genes (e.g., uricase gene). Under the synergistic action of *N. lugens* and yeast-like symbionts in the fat body, uric acid is finally degraded and reutilized by insect-yeast association [71,72]. The genome of endosymbiont *Dactylopiibacterium* mainly located in the ovaries of the carmine cochineal insects (*D. coccus*, *D. opuntiae*) was sequenced, and the data showed that purine and uric acid degrading genes were present in its genome [56]. In addition, fungal species (*Rhodotorula*, *Cryptococcus*, *Trametes*, *Penicillium*, *Debaryomyces*) associated with the carmine cochineal also exhibit urate oxidase activity in uric acid metabolism [73]. *Acyrthosiphon pisum* synergizes with the intracellular bacterium *Buchnera* through the host cell-mediated mode to assimilate ammonia from bacteriocyte metabolism into EAAs [15,74].

#### 4.2.5. Hymenoptera

Current research on NWR strategies in Hymenoptera mainly focuses on Formicidae insects. *Camponotus compressus* showed specific gustatory preferences for urea [75]. The overall transcriptional activity of urease structural gene *ureC*, urease accessory gene *ureF*, glutamine synthetase encoding gene *glnA* and arginase family encoding gene *speB* in the obligate intracellular endosymbiont *Blochmannia floridanus* increase steadily with carpenter ants age, which promotes the conversion of urea in chemically defined diets to all but arginine of the EAAs to the genus *Camponotus* [76,77]. Gene content within the gut microbiomes of 17 *Cephalotes* species showed that nearly all core symbionts involve in the biosynthesis of EAAs and nEAAs, but only a subset encode pathways of NWR, such as symbionts in Burkholderiales, Rhizobiales, and Opitutales. Burkholderiales mediate the degradation of purine, xanthine, or uric acid into urea, while Rhizobiales and Opitutales catalyze the conversion of urea into ammonia [20]. Similarly, gene function prediction in Bartonellaceae colonized in the midgut wall of *Dolichoderus* indicated that Bartonellaceae is capable of mediating nitrogen recycling and biosynthesis of several vitamins and all EAAs [78,79].

It should be stressed here that some insects on its own is sufficient for nitrogenous waste compounds degradation. For example, *Aedes aegypti* can efficiently incorporate ammonia into amino acids via GS/GOGAT cycle to reduce the toxicity of high-concentration ammonium salts to cells [80]. Urate oxidase encoding gene is actively expressed in the specific developmental stages of *Drosophila* (*D. melanogaster*, *D. pseudoobscura*, and *D. virilis*), and their nitrogenous metabolites are eventually excreted in the form of allantoin, allantoic acid, urea or ammonia [71,81]. However, a recent genomic investigation showed that the bacteria of the family Acetobacteraceae isolated from the guts of *Drosophila* fruit flies also possess the genes responsible for uric acid degradation [82]. Similar to *D. melanogaster*, the blowfly *Lucilia sericata* express high levels of urate oxidase in the malpighian tubules that convert uric acid from the hemolymph into allantoin that is excreted [81,83]. The uric acid that accumulates in silkworm tissues is excreted as a nitrogen waste product, but the genes of *Bmwh3*, *BmABCG5,* and *Bm5′N* encoded by silkworm are involved in uric acid metabolism [84]. Interestingly, urease in fresh mulberry leaves can directly pass through the gut wall of silkworm larvae into the hemolymph without being digested. Urea in midgut or hemolymph catalyzed by urease to ammonia is utilized by silkworm larvae to synthesize protein [85].

## 5. Discussion

BNF and NWR reactions can also function together within a given species, as observed in certain termites [86], cockroaches [87], bark beetles [65], longhorned beetles [30], and cochineal carmine [56,73], to compensate for extremely low dietary nitrogen levels. However, which strategy is more suitable for insects and what are the critical factors affecting strategies working remain largely unknown.

Here, the comparisons of two strategies may help us come up with the answers to the above questions. The similarities are as follows: (1) Both BNF and NWR pathways are mediated by symbionts, so any factors affecting the structure or expression level of the functional symbionts may affect the reaction efficiency of nitrogen cycle. For example, the state of nutrition affects the growth of functional symbionts. The interactions between insects and symbionts are enhanced to fix additional nitrogen source when the available nitrogen content is poor in diets. In contrast, a high-nitrogen diet inhibits the occurrence of BNF and NWR, or reduces the reaction efficiency [86]. (2) The end-product of the two strategies is ammonia, which is immediately assimilated by symbionts or insects, synthesizing nEAAs, EAAs, and other necessary nitrogenous metabolites via GS/GOGAT cycle.

On the other hand, the differences between BNF and NWR strategies are also evident: (1) Substrates for NWR reaction are diversified, as symbionts involved in this pathway are more abundant than that in BNF pathway. In the process of nitrogenous waste compounds degradation, functional genes encoding uricase, allantoinase, allantoicase, and urease are widely present in bacteria, fungi, and even host cells, whereas the nitrogenase complex only exists in some bacteria and the methanogens within Archaea [88], and most of nitrogen-fixing bacteria associated with insects belong to Enterobacteriaceae [42]. (2) These two strategies have different requirements for reaction conditions. Nitrogenase is extremely oxygen sensitive, due to Fe protein being rapidly inactivated by O_2_ [89], while the enzymatic reaction conditions mediating NWR strategy are relatively mild. (3) Energy consumption in BNF and NWR is different. Studies about BNF showed that two molecules of MgATP are required for each electron transfer, so at least 16 molecules of MgATP are consumed for each reduction of a dinitrogen [11,90]. By comparison, NWR strategy is more economical. Taking uric acid degradation in termite as an example, net energy consumption of the de novo synthesis of one molecule of uric acid only costs two molecules of MgATP. Furthermore, 11 additional molecules of MgATP can be produced during uric acid metabolism [48].

In summary, the nitrogenase system associated with insects is characterized by high energy consumption, complicated components, poor catalytic activity, and is extremely oxygen sensitive [10,90], which limits the general applicability of BNF strategy in insects, whereas NWR strategy is widespread in insect kingdom due to relatively mild reaction conditions, rich biodiversity of mediators, and the economical and efficient reaction process. In addition, we speculate that the available nitrogen content in diets is the decisive factor affecting the strategy choice of insects. Insects do not rely on BNF and NWR strategies to acquire nitrogen source if available nitrogen is abundant in diets. NWR strategy will be accelerated when nitrogen nutrition in diets cannot fully support the growth and development of insects. When the dietary nitrogen is extremely scarce and the environmental conditions for BNF reaction are met, the BNF reaction or a combination of multiple strategies may be accelerated for helping insects gain access to nitrogen nutrition.

## 6. Future Perspectives

Nitrogen acquisition strategies symbiotically mediated exist in insects widely, and provide insect–symbiont systems available nitrogen abundantly. Here, we propose the following points for future studies:

1. Existing research about BNF and NWR strategies mediated by insect symbionts is incomplete and very limited. The priority level (which strategy is more suitable for given insects), occurrence rules (which category of insects are more dependent on BNF or NWR), and characteristics (what is the critical factor for strategies working) of these two strategies in insects have not yet been revealed;
2. The extent of symbionts’ contribution to nitrogen provisioning in insects remains to be largely unknown. Specifically, how much nitrogen do insects fix through BNF or NWR? How much does this part of nitrogen nutrition account for the total insect demand? Addressing these questions will help us understand the significance of BNF and NWR during the coevolution between insects and symbionts;
3. Could the functional symbionts involved in BNF and NWR be exploited in mass rearing insects for production of bioprotein or empowering sterile insect technique (SIT)? The purpose is similar with the feeding pattern of “urea + ureolytic bacteria” in ruminants breeding. This pattern will achieve significant cost savings by transferring atmospheric nitrogen or nitrogenous waste into EAA/protein.

## Data Availability

Not applicable.

## Supplementary Materials

The following supporting information can be downloaded at: www.mdpi.com/article/10.3390/insects13010084/s1, Table S1: Evidence for the NWR pattern employed by some insects for nitrogen provisioning.
