# Supplementary material for: Nitrogen Acquisition Strategies Mediated by Insect Symbionts: A Review of Their Mechanisms, Methodologies, and Case Studies
Source: Insects. 2022 Jan 12;13(1):84. doi: 10.3390/insects13010084 (PMC8781418; doi:10.3390/insects13010084)
Supplement: Supplementary file 1 [file insects-13-00084-s001.zip › insects-1513106-supplementary.pdf]

Table S1. Evidence for the NWR pattern employed by some insects for nitrogen provisioning.

Insect	Nutritional supplies	developmental Stage	Functional bacteria	Bacteria Location	Nitrogenous waste	Methods	Reference
Blattaria: Cryptocercidae-*Periplaneta americana*	Omnivorous	Adult	*Blattabacterium cuenoti*	Fat body (Intracellular)	Ammonia, urea	Genomic analysis	[1, 2]
Blattaria: Cryptocercidae-*Periplaneta fuliginosa*	Omnivorous	Adult	*Blattabacterium* spp.	Fat body (Intracellular)	Ammonia, urea	Genomic analysis	[3]
Blattaria: Cryptocercidae-*Periplaneta japonica*	Omnivorous	Adult	*Blattabacterium* spp.	Fat body (Intracellular)	Ammonia, urea	Genomic analysis	[3]
Blattaria: Blattellidae-*Blattella germanica*	Omnivorous	Adult	*Blattabacterium cuenoti*	Fat body (Intracellular)	Ammonia, urea	Transcriptome analysis	[4]
Blattaria: Mastotermitidae-*Mastotermes*	Xylophagous	Adult	*Blattabacterium cuenoti*	Fat body; Hindgut	Ammonia, urea	Genomic analysis	[5, 6]

darwiniensis							
Blattaria: Rhinotermitidae-*Reticulitermes flavipes*	Xylophagous	Adult	*Streptococcus* sp.; *Bacteroides*; *Termitidis*; *Citrobacter*	Hindgut	Uric acid	Assimilation: ^{15}N-labeled uric acid; Biochemical tests (Enzyme assays)	[7, 8]
Blattaria: Rhinotermitidae-*Coptotermes formosanus*	Xylophagous	Adult	Bacteroidales	Gut	Ammonia, urea	Genomic analysis	[9]
Other wood-feeding termites (i.e. *Reticulitermes speratus*, *Coptotermes formosanus*,	Xylophagous	Adult	*Clostridia*; Enterobacteriaceae; Low G+C Gram-positive cocci	Gut	Uric acid	Uric acid-degrading bacteria isolation	[10]

Neotermes koshunensis, Glyptotermes fuscus, Cryptotermes domesticus, Hodotermopsis sjoestedti, Odontotermes formosanus, Nasutitermes takasagoensis)							
Coleoptera: Curculionidae-	Phloem	Adult; larva	*Serratia proteomaculans;*	Gut	Uric acid	Biochemical tests (Enzyme assays)	[11]

Dendroctonus rhizophagus			*Rahnella aquatilis*				
Coleoptera: Curculionidae-*Dendroctonus valens*	Phloem	Adult; larva	*Pseudomonas fluorescens; Serratia proteomaculans; Rahnella aquatilis*	Gut	Uric acid	Biochemical tests (Enzyme assays)	[11]
Coleoptera: Cerambycidae-*Anoplophora glabripennis*	Xylophagous	Larva; egg	Enterobacteriaceae	Gut	Urea	Assimilation: ^{15}N-labeled urea; Culture-independent approaches	[12]
Coleoptera: Scarabaeidae-*Melolontha hippocastani*	Root or leaf	Adult; larva	*Burkholderia; Parabacteroides*	Gut	Urea	Metagenome analysis; Assimilation: ^{15}N-labeled urea	[13]
Diptera: Stratiomyidae-*Hermetia illucens*	Organic waste	Larva	No highlight bacteria	Presumption in gut	Ammonia	Calculated biomass conversion ratio	[14]
Diptera: Tephritidae-*Bactrocera oleae*	Polyphagous	Adult female	*Candidatus* Erwinia dacicola	Gut	Urea	Monitored female fecundity	[15]
Diptera: Tephritidae-*Anastrepha ludens*	Polyphagous	Adult	*Enterobacter agglomerans*	Gut	Uric acid	Attraction assays	[16]
Diptera:	Rosaceae fruit	Adult	*Enterobacter*	Gut	Uric acid	Attraction assays	[17]

Tephritidae-*Rhagoletis pomonella*			*agglomerans*				
Diptera: Drosophilidae-*Drosophila melanogaster/ suzukii*	Polyphagous	Adult	*Acetobacter*	Gut	Uric acid	Genomic analysis; Biochemical tests (Enzyme assays)	[18]
Hemiptera: Aphididae-*Acyrthosiphon pisum*	Phloem sap	Larva	*Buchnera*	Bacteriocytes	Ammonia	Metabolite profile analysis	[19, 20]
Hemiptera: Parastrachiidae-*Parastrachia japonensis*	Monophagous (The drupes of *Shoepfia jasminodora*)	Nymph; adult	*Erwinia*-like bacteria	In the cecum of midgut	Uric acid	Biochemical tests (Enzyme assays)	[21]
Hemiptera: Delphacidae-*Nilaparvata lugens*	Monophagous (Rice sap)	Adult	Yeast-like symbionts	Intracellular	Uric acid	Biochemical tests (Enzyme assays);	[22, 23]
Hemiptera: Dactylopiidae-*Dactylopius coccus*	Cactus sap	Adult; egg; embryo	*Candidatus* Dactylopiibacterium carminicum; Uricolytic fungal: Rhodotorula, Cryptococcus,	Ovary;gut; egg surface	Uric acid	Genomic analysis; Biochemical tests (Enzyme assays); FISH	[24, 25]

			Trametes, Penicillium, Debaryomyces				
Hemiptera: Dactylopiidae-*Dactylopius opuntiae*	Cactus sap	Adult; egg; embryo	*Candidatus* Dactylopiibacterium carminicum; Uricolytic fungal: Rhodotorula, Cryptococcus, Trametes, Penicillium, Debaryomyces	Ovary;gut; egg surface	Uric acid	Genomic analysis; Biochemical tests (Enzyme assays); FISH	[24, 25]
Hymenoptera: Formicidae-*Camponotus floridanus*	Omnivorous	Adult; Larva; pupa; egg	*Blochmannia floridanus*	Bacteriocytes in midgut or ovary	Urea	Functional genes identification (*ureC*, *ureF*, *glnA*, and *speB*); Assimilation: ^{15}N-labeled urea	[26, 27]
Hymenoptera: Formicidae-*Camponotus compmssus*	Omnivorous	Adult	No highlight bacteria	Gut	Urea	Selection behaviors test	[28]
Hymenoptera: Formicidae-*Cephalotes varians*	Herbivorous	Adult	Burkholderiales; Opitutales; Rhizobiales	Gut	Urea, Uric acid	Metagenome analysis; Assimilation: ^{15}N-	[29]

						labeled urea	
Hymenoptera: Formicidae-*Cephalotes* species (i.e. *Cephalotes angustus*, *C. atratus*, *C. clypeatus*, *C. eduarduli*, *C. grandinosus*, *C. maculatus*, *C. minutus*, *C. pallens*, *C. pellans*, *C. persimilis*, *C. pusillus*, *C. rohweri*, *C. similimus*, *C. spinosus*, *C. umbraculatus*, *C. persimplex*)	Herbivorous	Adult	Burkholderiales; Opitutales; Rhizobiales	Gut	Urea, Uric acid	Metagenome analysis	[29]
Hymenoptera: Formicidae-*Dolichoderus* species	Plant exudates	Adult	Bartonellaceae	Midgut	Urea, uric acid	Genomic analysis	[30, 31]

1. Sabree, Z.L.; Kambhampati, S.; Moran, N.A. Nitrogen Recycling and Nutritional Provisioning by *Blattabacterium*, the Cockroach Endosymbiont. *P Natl Acad Sci USA* **2009**, *106*, 19521-19526, doi:10.1073/pnas.0907504106.
2. Mullins, D.E.; Cochran, D.G. Nitrogen-Metabolism in American Cockroach .1. An Examination of Positive Nitrogen-Balance with Respect to Uric-Acid Stores. *Comp Biochem Physiol* **1975**, *50*, 489-500, doi:10.1016/0300-9629(75)90306-0.
3. Vicente, C.S.L.; Mondal, S.I.; Akter, A.; Ozawa, S.; Kikuchi, T.; Hasegawa, K. Genome Analysis of New *Blattabacterium* Spp., Obligatory Endosymbionts of *Periplaneta Fuliginosa* and *P. Japonica*. *Plos One* **2018**, *13*, e0200512:1-e0200512:14, doi: 10.1371/journal.pone.0200512.
4. Patino-Navarrete, R.; Piulachs, M.D.; Belles, X.; Moya, A.; Latorre, A.; Pereto, J. The Cockroach *Blattella Germanica* Obtains Nitrogen from Uric Acid through a Metabolic Pathway Shared with Its Bacterial Endosymbiont. *Biol Letters* **2014**, *10*, doi:10.1098/rsbl.2014.0407.
5. Sabree, Z.L.; Huang, C.Y.; Arakawa, G.; Tokuda, G.; Lo, N.; Watanabe, H.; Moran, N.A. Genome Shrinkage and Loss of Nutrient-Providing Potential in the Obligate Symbiont of the Primitive Termite *Mastotermes Darwiniensis*. *Appl Environ Microb* **2012**, *78*, 204-210, doi:10.1128/Aem.06540-11.
6. Kinjo, Y.; Bourguignon, T.; Tong, K.J.; Kuwahara, H.; Lim, S.J.; Yoon, K.B.; Shigenobu, S.; Park, Y.C.; Nalepa, C.A.; Hongoh, Y.; etc. Parallel and Gradual Genome Erosion in the *Blattabacterium* Endosymbionts of *Mastotermes Darwiniensis* and *Cryptocercus* Wood Roaches. *Genome Biol Evol* **2018**, *10*, 1622-1630, doi:10.1093/gbe/evy110.
7. Potrikus, C.J.; Breznak, J.A. Gut Bacteria Recycle Uric-Acid Nitrogen in Termites - a Strategy for Nutrient Conservation. *P Natl Acad Sci-Biol* **1981**, *78*, 4601-4605, doi:10.1073/pnas.78.7.4601.
8. Potrikus, C. J.; Breznak, J. A. Uric Acid- Degrading Bacteria in Guts of Termites [*Reticulitermes Flavipes* (Kollar)]. *Appl Environ Microb* **1980**, *40*, 117-124, doi:10.1128/Aem.40.1.117-124.1980.
9. Hongoh, Y.; Sharma, V.K.; Prakash, T.; Noda, S.; Toh, H.; Taylor, T.D.; Kudo, T.; Sakaki, Y.; Toyoda, A.; Hattori, M.; etc. Genome of an Endosymbiont Coupling N_2 Fixation to Cellulolysis within Protist Cells in Termite Gut. *Science* **2008**, *322*, 1108-1109, doi:10.1126/science.1165578.
10. Thong-On, A.; Suzuki, K.; Noda, S.; Inoue, J.; Kajiwara, S.; Ohkuma, M. Isolation and Characterization of Anaerobic Bacteria for Symbiotic Recycling of Uric Acid Nitrogen in the Gut of Various Termites. *Microbes Environ* **2012**, *27*, 186-192, doi:10.1264/jsme2.ME11325.
11. Morales-Jimenez, J.; de Leon, A.V.P.; Garcia-Dominguez, A.; Martinez-Romero, E.; Zuniga, G.; Hernandez-Rodriguez, C. Nitrogen-Fixing and Uricolytic Bacteria Associated with the Gut of *Dendroctonus Rhizophagus* and *Dendroctonus Valens* (Curculionidae:

Scolytinae). *Microb Ecol* **2013**, *66*, 200-210, doi:10.1007/s00248-013-0206-3.
12. Ayayee, P.; Rosa, C.; Ferry, J.G.; Felton, G.; Saunders, M.; Hoover, K. Gut Microbes Contribute to Nitrogen Provisioning in a Wood-Feeding Cerambycid. *Environ Entomol* **2014**, *43*, 903-912, doi:10.1603/En14045.
13. Alonso-Pernas, P.; Bartram, S.; Arias-Cordero, E.M.; Novoselov, A.L.; Halty-deLeon, L.; Shao, Y.Q.; Boland, W. In Vivo Isotopic Labeling of Symbiotic Bacteria Involved in Cellulose Degradation and Nitrogen Recycling within the Gut of the Forest Cockchafer (*Melolontha Hippocastani*). *Front Microbiol* **2018**, *9*, 488:1-488:15, doi:10.3389/fmicb.2018.00488.
14. Isibika, A.; Vinneras, B.; Kibazohi, O.; Zurbrugg, C.; Lalander, C. Pre-Treatment of Banana Peel to Improve Composting by Black Soldier Fly (*Hermetia Illucens* (L.), Diptera: Stratiomyidae) Larvae. *Waste Manage* **2019**, *100*, 151-160, doi:10.1016/j.wasman.2019.09.017.
15. Ben-Yosef, M.; Pasternak, Z.; Jurkevitch, E.; Yuval, B. Symbiotic Bacteria Enable Olive Flies (*Bactrocera Oleae*) to Exploit Intractable Sources of Nitrogen. *J Evolution Biol* **2014**, *27*, 2695-2705, doi:10.1111/jeb.12527.
16. Robacker, D.C.; Lauzon, C.R. Purine Metabolizing Capability of *Enterobacter Agglomerans* Affects Volatiles Production and Attractiveness to Mexican Fruit Fly. *J Chem Ecol* **2002**, *28*, 1549-1563, doi:10.1023/A:1019920328062.
17. Lauzon, C.R.; Sjogren, R.E.; Prokopy, R.J. Enzymatic Capabilities of Bacteria Associated with Apple Maggot Flies: A Postulated Role in Attraction. *J Chem Ecol* **2000**, *26*, 953-967, doi:10.1023/A:1005460225664.
18. Winans, N.J.; Walter, A.; Chouaia, B.; Chaston, J.M.; Douglas, A.E.; Newell, P.D. A Genomic Investigation of Ecological Differentiation between Free-Living and Drosophila-Associated Bacteria. *Mol Ecol* **2017**, *26*, 4536-4550, doi:10.1111/mec.14232.
19. Macdonald, S.J.; Lin, G.G.; Russell, C.W.; Thomas, G.H.; Douglas, A.E. The Central Role of the Host Cell in Symbiotic Nitrogen Metabolism. *P Roy Soc B-Biol Sci* **2012**, *279*, 2965-2973, doi:10.1098/rspb.2012.0414.
20. Hansen, A.K.; Moran, N.A. Aphid Genome Expression Reveals Host-Symbiont Cooperation in the Production of Amino Acids. *P Natl Acad Sci USA* **2011**, *108*, 2849-2854, doi:10.1073/pnas.1013465108.
21. Kashima, T.; Nakamura, T.; Tojo, S. Uric Acid Recycling in the Shield Bug, *Parastrachia Japonensis* (Hemiptera : Parastrachiidae), During Diapause. *J Insect Physiol* **2006**, *52*, 816-825, doi:10.1016/j.jinsphys.2006.05.003.
22. Hongoh, Y.; Sasaki, T.; Ishikawa, H. Cloning, Sequence Analysis and Expression in *Escherichia Coli* of the Gene Encoding a Uricase from the Yeast-Like Symbiont of the Brown Planthopper, *Nilaparvata Lugens*. *Insect Biochem Molec* **2000**, *30*, 173-182, doi:10.1016/S0965-1748(99)00116-2.
23. Xue, J.; Zhou, X.; Zhang, C.X.; Yu, L.L.; Fan, H.W.; Wang, Z.; Xu, H.J.; Xi, Y.; Zhu, Z.R.; Zhou, W.W.; etc. Genomes of the Rice Pest

Brown Planthopper and Its Endosymbionts Reveal Complex Complementary Contributions for Host Adaptation. *Genome Biol* **2014**, *15*, 521:1-521:20, doi:10.1186/s13059-014-0521-0.
24. de Leon, A.V.P.; Ormeno-Orrillo, E.; Ramirez-Puebla, S.T.; Rosenblueth, M.; Esposti, M.D.; Martinez-Romero, J.; Martinez-Romero, E. *Candidatus* Dactylopiibacterium Carminicum, a Nitrogen-Fixing Symbiont of *Dactylopius* Cochineal Insects (Hemiptera: Coccoidea: Dactylopiidae). *Genome Biol Evol* **2017**, *9*, 2237-2250, doi:10.1093/gbe/evx156.
25. de Leon, A.V.P.; Sanchez-Flores, A.; Rosenblueth, M.; Martinez-Romero, E. Fungal Community Associated with *Dactylopius* (Hemiptera: Coccoidea: Dactylopiidae) and Its Role in Uric Acid Metabolism. *Front Microbiol* **2016**, *7*, 954:1-954:15, doi:10.3389/fmicb.2016.00954.
26. Zientz, E.; Beyaert, N.; Gross, R.; Feldhaar, H. Relevance of the Endosymbiosis of *Blochmannia Floridanus* and Carpenter Ants at Different Stages of the Life Cycle of the Host. *Appl Environ Microb* **2006**, *72*, 6027-6033, doi:10.1128/Aem.00933-06.
27. Feldhaar, H.; Straka, J.; Krischke, M.; Berthold, K.; Stoll, S.; Mueller, M.J.; Gross, R. Nutritional Upgrading for Omnivorous Carpenter Ants by the Endosymbiont *Blochmannia*. *Bmc Biol* **2007**, *5*, 48:1-48:11, doi:10.1186/1741-7007-5-48.
28. Shetty, P.S. Gustatory Preferences of Ants (*Camponotus-Compressus*) for Urea and Sugars. *Experientia* **1982**, *38*, 259-260, doi:10.1007/Bf01945100.
29. Hu, Y.; Sanders, J.G.; Lukasik, P.; D'Amelio, C.L.; Millar, J.S.; Vann, D.R.; Lan, Y.M.; Newton, J.A.; Schotanus, M.; Kronauer, D.J.C.; et al. Herbivorous Turtle Ants Obtain Essential Nutrients from a Conserved Nitrogen-Recycling Gut Microbiome. *Nat Commun* **2018**, *9*, 2440:1-2440:14, doi:10.1038/s41467-018-04935-w.
30. Bisch, G.; Neuvonen, M.M.; Pierce, N.E.; Russell, J.A.; Koga, R.; Sanders, J.G.; Lukasik, P.; Andersson, S.G.E. Genome Evolution of Bartonellaceae Symbionts of Ants at the Opposite Ends of the Trophic Scale. *Genome Biol Evol* **2018**, *10*, 1687-1704, doi:10.1093/gbe/evy126.
31. Cook, S.C.; Davidson, D.W. Nutritional and Functional Biology of Exudate-Feeding Ants. *Entomol Exp Appl* **2006**, *118*, 1-10, doi:10.1111/j.1570-7458.2006.00374.x.
